# Anesthesia management for laparoscopic surgery in the Trendelenburg position in a patient with Fontan physiology and severe hypoxemia: a case report

**DOI:** 10.1186/s40981-025-00820-w

**Published:** 2025-09-29

**Authors:** Shunsuke Okano, Masahiro Kuroki, Shun Abe, Yu Matsuura, Ayuka Narisawa, Hiroaki Toyama

**Affiliations:** Department of Anesthesiology, Faculty of Medicine, Yamagata University, 2-2-2 Iida-Nishi, Yamagata, 990-9585 Japan

**Keywords:** Fontan physiology, Laparoscopic surgery, Trendelenburg position, Hypoxemia, Case report

## Abstract

**Background:**

Pneumoperitoneum and Trendelenburg positioning are thought to increase pulmonary vascular resistance (PVR). In Fontan circulation, increased PVR is directly related to decreased ventricular preload and can result in circulatory failure.

**Case presentation:**

A 23-year-old female patient with Fontan physiology was diagnosed with left paraovarian cyst torsion and underwent laparoscopic cystectomy. SpO_2_ was 70% in room air. General anesthesia was induced with remimazolam, fentanyl, and rocuronium and maintained with remimazolam and remifentanil combined with an abdominal wall block. The difference between SpO_2_ and central venous oxygen saturation (ScvO_2_) increased during the induction of anesthesia and further increased with the initiation of pneumoperitoneum and Trendelenburg positioning but recovered at the completion of the surgery.

**Conclusions:**

Patients with Fontan physiology and severe hypoxemia can tolerate short-term laparoscopic surgery in the Trendelenburg position under strict management. In these patients, monitoring ScvO_2_ provides important circulatory information regarding the effects of pneumoperitoneum and Trendelenburg positioning.

## Background

Recent improvements in the diagnostic and therapeutic techniques for congenital heart disease have resulted in an increase in long-term survival of patients with Fontan physiology [1]. An increase in the number of adult congenital heart disease cases has also increased the number of patients with Fontan physiology, and more of these patients are undergoing noncardiac surgeries [2]. Because Fontan circulation lacks right ventricular contraction, maintaining adequate central venous pressure (CVP) and low pulmonary vascular resistance (PVR) is important for blood circulation [3]. Positive pressure ventilation increases PVR by increasing pulmonary capillary pressure [4]. During laparoscopic surgery, PVR can increase owing to increased intrathoracic pressure associated with diaphragmatic elevation, increased atelectasis, and increased arterial partial carbon dioxide (CO_2_) tension (PaCO_2_) by the absorption of CO_2_ by the pneumoperitoneum [5]. In Fontan physiology, the driving pressure for pulmonary circulation is CVP; therefore, an elevated PVR impairs pulmonary circulation, resulting in venous congestion, hypotension due to decreased ventricular preload reserve, and eventual circulatory failure.

Herein, we present a patient with Fontan circulation and severe hypoxemia who underwent general anesthesia for laparoscopic paraovarian cystectomy in the Trendelenburg position. In this case, comparing central venous oxygen saturation (ScvO_2_) and SpO_2_ was useful for management. This case report follows the CARE guidelines [6] and the Anaesthesia Case Report checklist [7].

## Case presentation

**Fig. 1:**
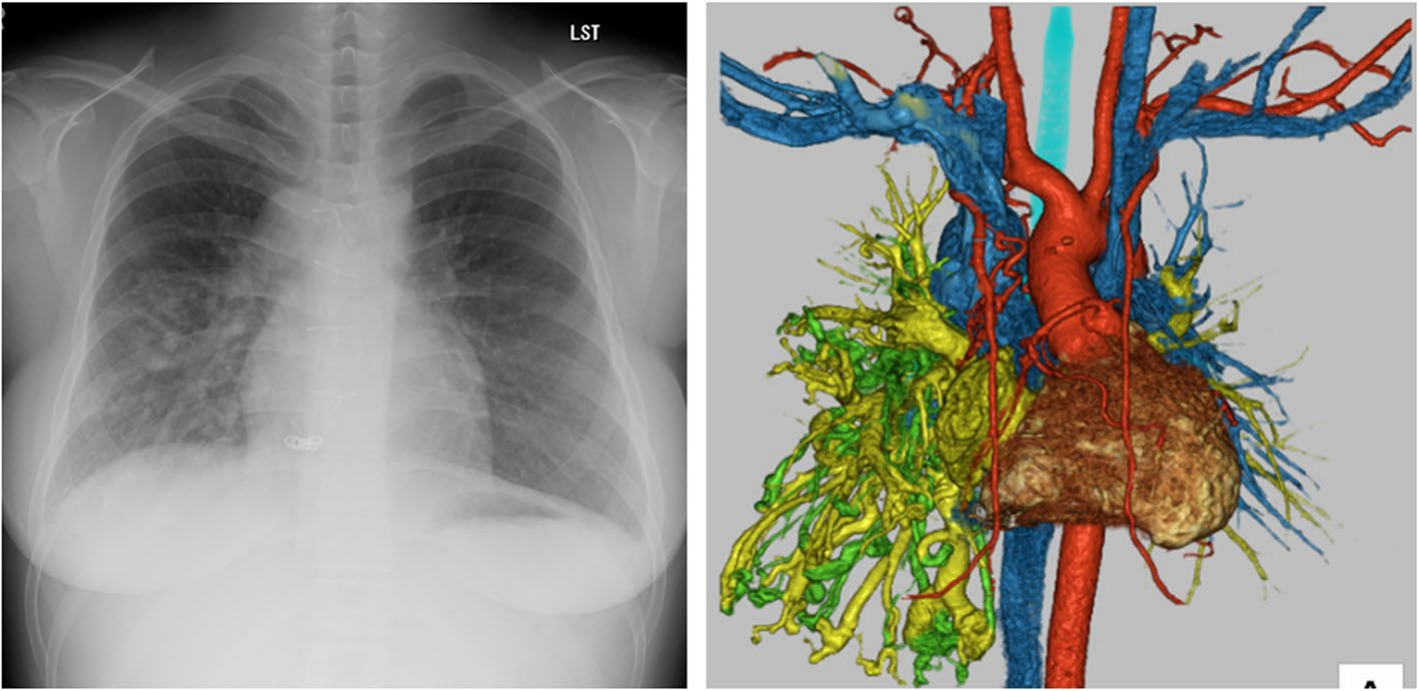
Preoperative chest images. Chest radiography taken 4 days before the operation (left) and three-dimensional computed tomography angiography (right) showing increased vascular shadows in the right lung field and presence of a pulmonary arteriovenous fistula, respectively. The red structures represent arteries, the blue structures represent veins and the left pulmonary arteries, the green structures represent the right pulmonary arteries, and the yellow structures represent pulmonary veins and the left atrium

The patient was a 23-year-old female (height 149 cm, weight 53 kg). She was diagnosed at birth with polysplenia, a double-outlet right ventricle, an unbalanced atrioventricular septal defect, pulmonary artery stenosis, partial anomalous pulmonary venous return, and azygos continuation. The patient underwent a left Blalock-Taussig shunt at the age of 1 year and lateral tunnel total cavopulmonary bypass surgery at the age of 3 years. A fenestration was not created at the time of the latter surgery. Many pulmonary arteriovenous fistulas (PAVF) were noted in the right lung (Fig. 1), and the patient presented with central cyanosis and class II of the New York Heart Association functional class.

**Fig. 2:**
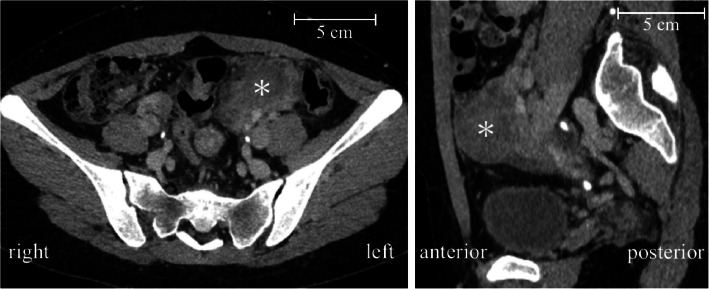
Preoperative abdominal computed tomography images. Left paraovarian cyst torsion (*) is visible in the coronal pelvic image (left). The sagittal abdominal image (right) shows the cyst (diameter 34 × 43 mm)

The patient visited the hospital with complaints of abdominal pain and was diagnosed with left paraovarian cyst torsion, leading to a course of semi-emergent surgery (Fig. 2). Admission blood pressure (BP) was 110/58 mmHg, and the heart rate was 101 beats/min with sinus rhythm. The SpO_2_ in room air was 77%, hemoglobin level was 20.4 g/dL, and hematocrit was 63.9% (Table 1). Echocardiography showed preserved ventricular contractions and mild atrioventricular valve regurgitation. Cardiac catheterization 6 years prior revealed a CVP of 16 mmHg, pulmonary artery wedge pressure of 12 mmHg, cardiac output (CO) of 2.3 L/min, pulmonary to systemic flow ratio of 0.6, PVR index of 2.8 WU*m^2^, and a ventricular ejection fraction of 61%.

**Table 1:** Perioperative blood gas analysis of the patient

Time points	Before anesthesia induction		After recovery from anesthesia
Sampling site	Artery	Superior vena cava	Artery
Inhalational oxygen	Room air		3 L/min
pH	7.230	7.393	7.371
PCO_2_ (mmHg)	29.9	38.5	37.8
PO_2_ (mmHg)	43.7	37.5	54.1
HCO_3_^−^ (mmol/L)	19.0	22.9	21.4
Base excess (mmol/L)	−3.7	−1.5	−3.2
SO_2_ (%)	77.1	62.9	85.1
Hemoglobin (g/dL)	20.0	20.6	20.1
Hematocrit (%)	59	61	59
Lactate (mmol/L)	0.91	0.94	0.79

*pH* Hydrogen potential, *PCO*_*2*_ Partial carbon dioxide tension, *PO*_*2*_ Partial oxygen tension, *HCO*_*3*_^*−*^ bicarbonate, *SO*_*2*_ Oxygen saturation

The surgeon planned a laparoscopic paraovarian cystectomy requiring pneumoperitoneum and Trendelenburg positioning. Although the patient's elevated CVP and PVR suggested that the procedure could potentially cause circulatory failure, laparoscopic surgery was deemed feasible because the ventricular ejection fraction was relatively preserved and the PAVF might prevent excessive CVP elevation and reduction in ventricular preload.

**Fig. 3:**
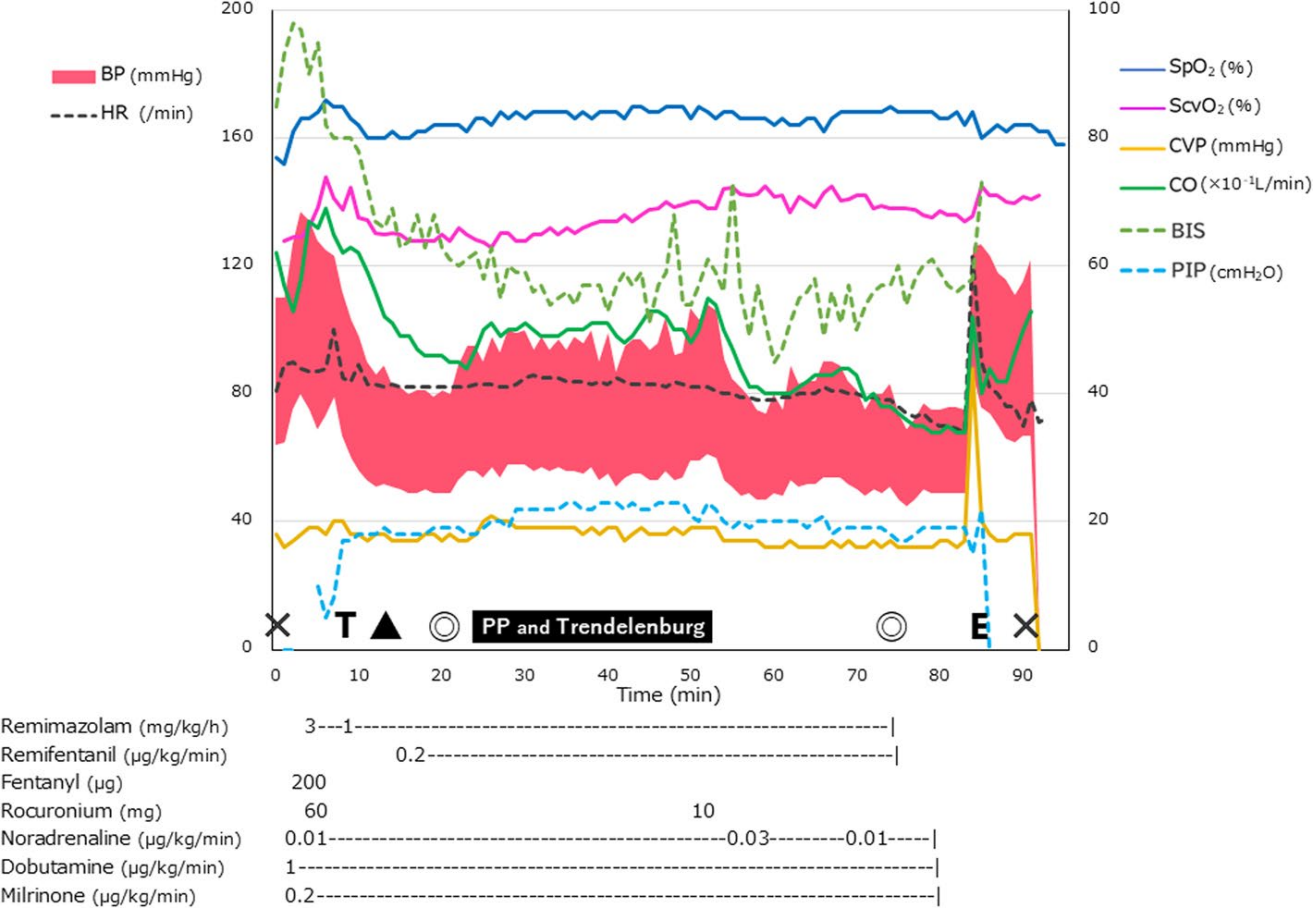
Changes of hemodynamic parameters during anesthesia. During the induction of anesthesia, blood pressure (BP) and cardiac output (CO) decreased, and the difference between percutaneous oxygen saturation (SpO_2_) and central venous oxygen saturation (ScvO_2_) (SpO_2_-ScvO_2_) increased to 18%. When pneumoperitoneum was initiated in the Trendelenburg position, BP and central venous pressure (CVP) increased mildly, and SpO_2_-ScvO_2_ increased temporarily but then began to decrease. Postoperatively, BP decreased significantly, CVP decreased mildly, and the noradrenaline dose was increased. The SpO_2_-ScvO_2_ decreased to approximately 13%. After awakening, the SpO_2_-ScvO_2_ difference decreased to 8% owing to increased CO. BP, blood pressure; HR, heart rate; SpO_2_, percutaneous oxygen saturation; ScvO_2_, central venous oxygen saturation; CVP, central venous pressure; CO, cardiac output; BIS, bispectral index; PIP, peak inspiratory pressure; ×, start or end of anesthesia; T, tracheal intubation; ▲, transversus abdominis plane block; ⊚, start or end of surgery; PP and Trendelenburg, duration of pneumoperitoneum and Trendelenburg position; and E, tracheal extubation

In the operating room, an oximetric central venous catheter (PreSep Catheter™; Edwards Lifesciences Corporation, Irvine, CA, USA) was placed via the right internal jugular vein. An arterial catheter was placed in the left radial artery, and arterial pressure-based cardiac output (APCO) (FloTrac Sensor™; Edwards Lifesciences) was also monitored. Blood gas analysis revealed an arterial oxygen saturation of 77.1% and ScvO_2_ of 62.9%, a difference of 14.2% (Table 1). During preoxygenation, noradrenaline 0.01 µg/kg/min, dobutamine 1.0 µg/kg/min, and milrinone 0.2 µg/kg/min were initiated (Fig. 3). Subsequently, general anesthesia was induced with remimazolam 3 mg/kg/h, fentanyl 200 µg, and rocuronium 60 mg, and an ID 7.0 mm tracheal tube was intubated. Pressure-regulated volume control ventilation was initiated with a tidal volume of 400 mL, a respiratory rate 18 breaths/min, and positive end-expiratory pressure of 5 cmH_2_O. The end-tidal CO_2_ tension was maintained at approximately 33 torr. During this period, BP and APCO decreased, and the difference between SpO_2_ and ScvO_2_ increased to 18% (Fig. 3). However, these physiological effects were considered minimal, and anesthesia was continued with remimazolam 1 mg/kg/h and remifentanil 0.2 µg/kg/min. A transversus abdominis plane block was performed with 50 mL of 0.2% levobupivacaine. Five minutes after the start of surgery, the laparoscopic procedure was started and the patient was placed in the Trendelenburg position at approximately 5°, leading to mildly elevated BP and CVP. The SpO_2_-ScvO_2_ difference temporarily increased to 21%, but gradually recovered to 15% (Fig. 3). Laparoscopy revealed a hemorrhagic left paraovarian cyst. Adhesiolysis and tissue biopsies were performed. Upon completion of the laparoscopy, a significant decrease in BP and mild decrease in CVP were observed; therefore, the noradrenaline infusion was increased. The SpO_2_-ScvO_2_ difference decreased to approximately 13% (Fig. 2). Ten minutes postoperatively, sugammadex 200 mg and flumazenil 0.4 mg were administered. After recovery of the train of four ratio to 105% and spontaneous breathing, the patient was awakened and subsequently extubated. Soon thereafter, the SpO_2_-ScvO_2_ difference decreased to 8% owing to increased CO (Fig. 2). Arterial blood gas analysis revealed a slight increase in the partial pressure of oxygen (PaO_2_) with oxygen inhalation and an increase in PaCO_2_ with mild respiratory depression. However, no decrease in base excess or increase in lactate was noted (Table 1). Little bleeding and 150 mL of urine volume during anesthesia; 800 mL of crystalloids was infused.

The patient was transferred to a high-care unit, returned to the general ward on the first postoperative day, and discharged on the fifth postoperative day without complications.

## Discussion

Searching for the keywords “Fontan” and “laparoscopic” in literature search engines, including PubMed (accessed August 31, 2025), revealed that this case had the lowest SpO_2_ among Fontan patients undergoing laparoscopic surgery. Furthermore, this is the third reported case of laparoscopic surgery in the Trendelenburg position in a patient with Fontan circulation [8, 9].

In patients with Fontan physiology, preoperative hypoxemia is associated with a significant increase in intraoperative and postoperative adverse events [10]. Our literature search revealed only one case report of a patient with Fontan physiology and multiple PAVF, like our case, whose surgery was terminated owing to worsening hypoxemia and heart failure during pneumonectomy [11]. However, limited reports have been published on laparoscopic procedures in patients with Fontan physiology. Therefore, predicting how pneumoperitoneum, changes in inspired oxygen, and changes in the Trendelenburg position affect the blood flow of the PAVF and hypoxemia is difficult. To distinguish between the causes of intraoperative hypoxemia and cardiac failure, we used oximetry, central venous catheters, APCO, and transthoracic echocardiography. Worsening hypoxemia accompanied by decreased APCO is considered cardiogenic hypoxemia, whereas worsening hypoxemia accompanied by maintained APCO is considered an increase in PAVF blood flow or intrapulmonary shunting caused by such conditions as atelectasis or pulmonary edema. An unchanging APCO and an increase in the SpO_2_-ScvO_2_ difference may indicate increased oxygen consumption or decreased hemoglobin concentration. During general anesthesia under positive-pressure ventilation, the PVR increases with an increase in pulmonary capillary pressure [4]. In our case, cardiac depression induced by anesthetics and positive-pressure ventilation caused a decrease in BP and APCO, with a concomitant increase in the difference between SpO_2_ and ScvO_2_.

Insufflation of CO_2_ in pneumoperitoneum increases PVR [5]. Trendelenburg positioning can elevate the diaphragm, increase atelectasis, and further increase the PVR [5, 12]. Particularly in patients after the Fontan procedure, the increased PVR may impair maintenance of the ventricular preload reserve, resulting in a more pronounced decrease in CO [13, 14]. Conversely, increasing CVP by increasing venous return may be favorable for preserving the preload reserve [5]. Actually, previous reports have not shown any adverse events associated with the Trendelenburg position [8, 9]. The advantages of laparoscopic surgery include reduced wound pain, better cosmetic appearance, and decreased blood loss [15, 16]. Reduced wound pain may decrease sympathetic excitation, thereby decreasing the incidence of arrhythmias and the deterioration of respiratory function. Additionally, early postoperative ambulation may be possible, which may reduce the incidence of thrombosis. Considering these advantages and disadvantages, laparoscopic surgery was chosen in the present case. Trendelenburg positioning and pneumoperitoneum caused mild increases in BP, CVP, and CO in our patient, whereas the difference between SpO_2_ and ScvO_2_ increased. This may have been caused by increased oxygen consumption from peritoneal stimulation or sympathetic excitation facilitated by the pneumoperitoneum. The difference eventually decreased, possibly owing to adaptation to the condition and a decrease in oxygen consumption, or because the hemodilution associated with anesthesia induction was ameliorated by redistribution and urination. At the resolution of the pneumoperitoneum, the difference decreased further. More cases are required to gain insight into whether prolonged Trendelenburg positioning and pneumoperitoneum widen the difference between SpO_2_ and ScvO_2_. In our case, laparoscopy with the patient in Trendelenburg positioning took a relatively short time, was well tolerated, and no adverse events occurred. However, not all patients with Fontan physiology can undergo laparoscopy safely. The long-term condition of patients after Fontan surgery varies significantly [17, 18]. Anesthesiologists managing patients with Fontan physiology must have a thorough understanding and knowledge of the characteristics of Fontan physiology and individual case differences. Collectively, the entire surgical team should make comprehensive decisions on the best operative procedures and anesthesia management for patients.

## Data Availability

Data relevant to this case report is not available for public access because of patient privacy concerns; however, it is available from the corresponding author upon reasonable request.

## Abbreviations

APCO: Arterial pressure-based cardiac output
BP: Blood pressure
CO: Cardiac output
CO_2_: Carbon dioxide
CVP: Central venous pressure
PaCO_2_: Arterial partial carbon dioxide tension
PAVF: Pulmonary arteriovenous fistula
PVR: Pulmonary vascular resistance
ScvO_2_: Central venous oxygen saturation
SpO_2_: Percutaneous oxygen saturation
